# Antiphospholipid Syndrome Mimicking Acute Exacerbation of Interstitial Pneumonia: A Case Report and Literature Review

**DOI:** 10.1002/rcr2.70473

**Published:** 2026-01-14

**Authors:** Saki Ishii, Hiroki Wakabayashi, Kazutoshi Isobe, Ryogo Ohashi, Kensuke Namba, Misa Iwayanagi, Hiromasa Sakurai, Daiki Sakai, Kenta Takashima, Yu Murakami, Kaichi Kaneko, Yasuo Matsuzawa

**Affiliations:** ^1^ Division of Respiratory Medicine, Department of Internal Medicine Toho University Sakura Medical Center Chiba Japan

**Keywords:** acute exacerbation of interstitial pneumonia, anticoagulation therapy, antiphospholipid syndrome, autoimmune disease, catastrophic antiphospholipid syndrome

## Abstract

Acute exacerbation of interstitial pneumonia (IP‐AE) is a type of severe respiratory failure that occurs in patients with chronic interstitial pneumonia. Herein, we report a case of multiple pulmonary thrombi caused by antiphospholipid antibody syndrome (APS), which required differentiation from IP‐AE in a patient with chronic interstitial pneumonia. A 77‐year‐old male patient presented with acute respiratory failure and bilateral ground‐glass opacities on chest computed tomography (CT), which initially indicated IP‐AE. However, the contrast‐enhanced CT scan revealed multiple pulmonary thrombi, and the laboratory examination showed positivity for antiphospholipid antibodies. The patient was diagnosed with APS and was successfully treated with anticoagulant therapy and systemic corticosteroids. The pulmonary manifestations of APS may mimic those of IP‐AE and may be under‐recognised. Thus, APS should be considered in the differential diagnosis of acute respiratory deterioration in patients with interstitial pneumonia.

## Graphical abstract

Acute exacerbation of interstitial pneumonia (IP‐AE) is a type of severe respiratory failure that occurs in patients with chronic interstitial pneumonia. However, a few cases of lung involvement caused by antiphospholipid antibody syndrome (APS) appearing similar to IP‐AE have been reported. Here, we present a case of APS manifesting as IP‐AE in which the patient achieved a favorable outcome with initial combination therapy using corticosteroids and anticoagulants followed by maintenance anticoagulation, and provide a literature review.

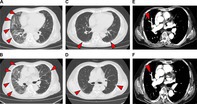

## 1 Introduction

Acute exacerbation of interstitial pneumonia (IP‐AE) is a life‐threatening condition that occurs in patients with chronic interstitial pneumonia. IP‐AE is characterised by rapid worsening of respiratory symptoms, new bilateral ground‐glass opacities on chest computed tomography (CT) scan [1, 2] and a high mortality rate reaching nearly 50% [2]. The common triggers include infections, surgical procedures, bronchoscopy, use of certain medications, aspiration, dust exposure and smoking [1]. However, in several cases, the underlying cause remains unclear.

Antiphospholipid syndrome (APS) is an autoimmune disorder characterised by the presence of antiphospholipid antibodies, which lead to thrombosis and organ dysfunction [3, 4]. Its diagnosis requires both clinical evidence of thrombosis and persistent positivity for lupus anticoagulant, anticardiolipin antibodies or anti‐β2 glycoprotein I antibodies on two occasions, at least 12 weeks apart [4, 5]. Severe cases are classified as catastrophic antiphospholipid syndrome (CAPS), which can cause life‐threatening pulmonary complications. The standard treatment for APS is anticoagulation therapy, which is also utilised in cases of severe pulmonary involvement attributed to CAPS [6]. However, the association between interstitial pneumonia and APS remains poorly understood.

Herein, we report a case of APS with pulmonary involvement that required differentiation from IP‐AE. Treatment with anticoagulant drugs and systemic corticosteroids resulted in a favourable outcome. Differentiating APS‐associated pulmonary involvement from IP‐AE is essential for appropriate management.

## 2 Case Report

A 77‐year‐old male patient was referred to our hospital for the treatment of IP‐AE. He previously visited another hospital due to a chief complaint of dyspnea for 3 days. He was diagnosed with IP‐AE. Further, he was hospitalised because of respiratory failure, as evidenced by an oxygen saturation of 80% on room air, abnormal shadows on chest radiography and CT scan, and elevated serum Krebs von den Lungen‐6 levels. The patient was transported to our hospital the day after admission at the previous hospital for further treatment. Six years earlier, abnormal chest shadows had been detected during a routine health check‐up, and the patient was subsequently diagnosed with interstitial pneumonia at our institution.

**FIGURE 1:**
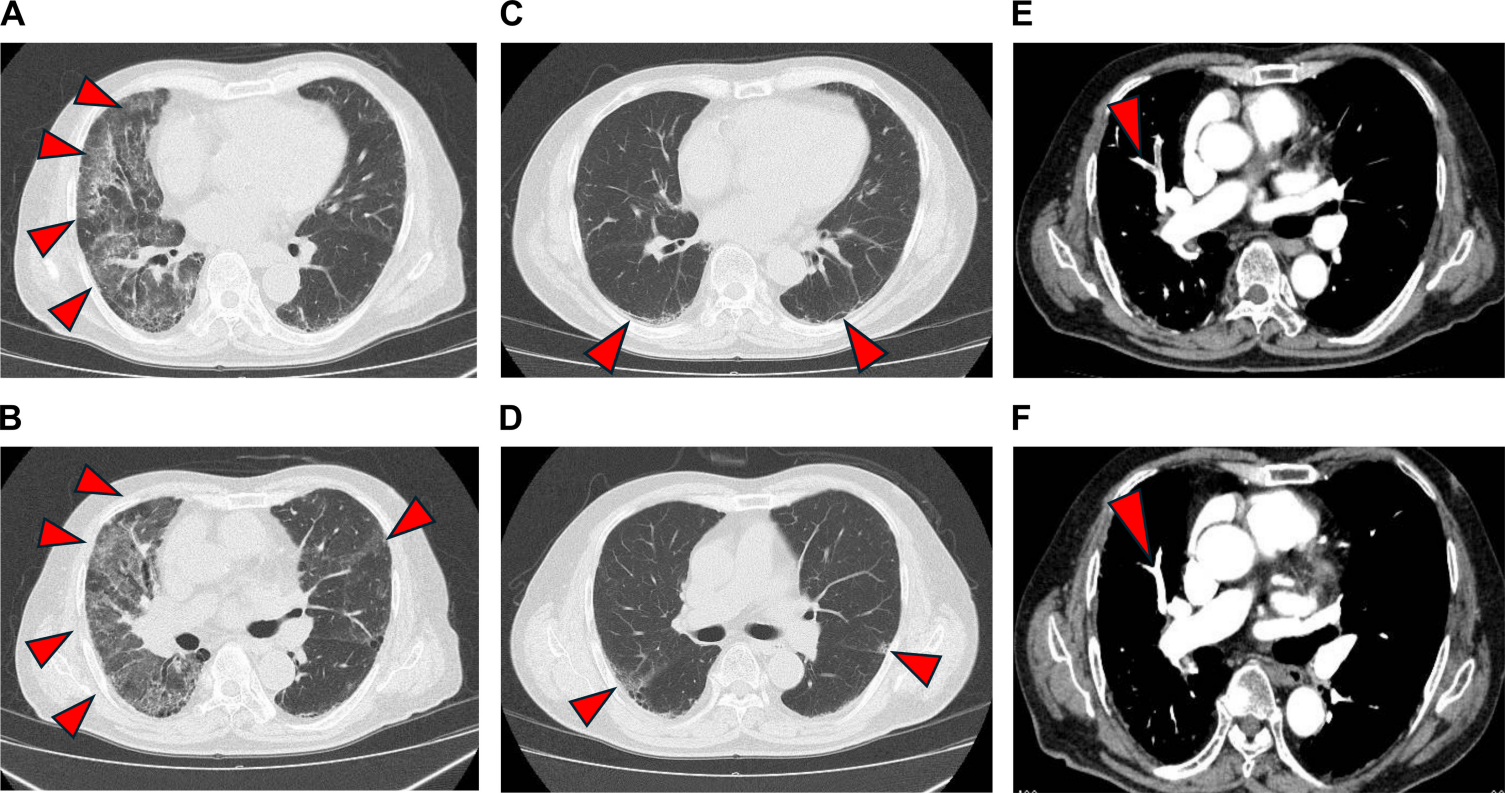
Chest CT scan showed ground‐glass opacities in the bilateral lung fields (A, B), which were not observed 6 years back (C, D). Ground‐glass opacities and reticular shadows in the bilateral peripheral lung field, suspected to be signs of chronic interstitial pneumonia, were observed (D, arrows). Contrast‐enhanced CT scan revealed multiple contrast‐enhanced defects mainly in the peripheral lower lobe branches of the bilateral pulmonary arteries (E, arrows). Contrast‐enhanced CT scan performed 1 month after discharge showed that the pulmonary thrombus had improved (F, arrows).

**FIGURE 2:**
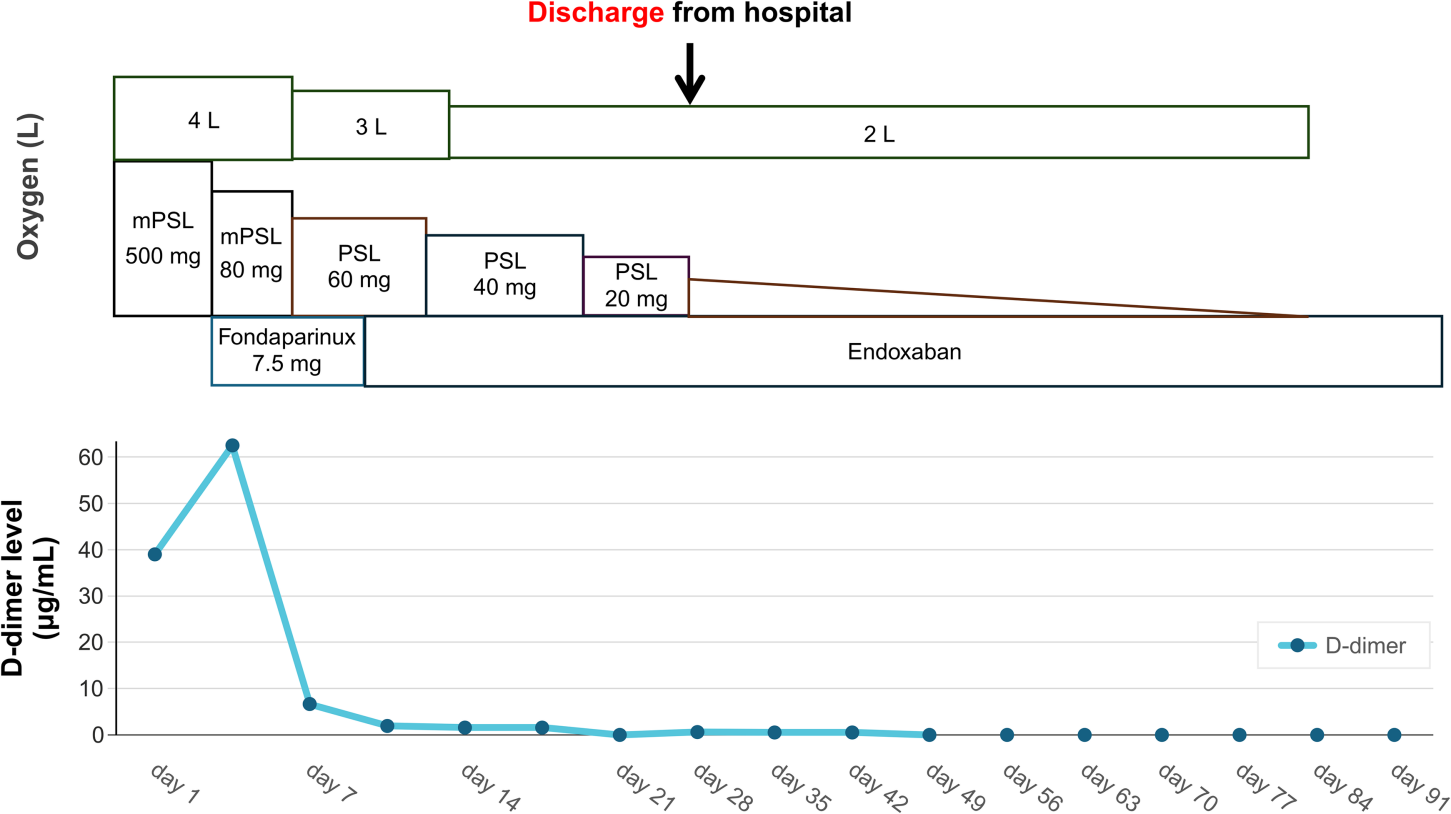
Clinical course of the patient. mPSL, methyl prednisolone; PSL, prednisolone.

Physical examination revealed the following findings: body temperature, 36.9°C; blood pressure, 111/70 mmHg; pulse rate, 66 beats/min; respiratory rate, 28 cycles/min; and oxygen saturation, 95% on 2 L/min oxygen. Auscultation confirmed fine crackles on the bilateral anterior lungs. The laboratory tests showed elevated C‐reactive protein (7.08 mg/dL), lactate dehydrogenase (360 IU/L), Krebs von den Lungen‐6 (1236 U/mL) and D‐dimer (38.97 μg/mL) levels (Table 1). Chest radiography revealed attenuation in the right upper lung field, and chest CT scan found ground‐glass opacities in the bilateral lung fields (Figure 1A,B), which were not observed 6 years back (Figure 1C,D). The electrocardiogram and transthoracic echocardiography results were normal. The polymerase chain reaction for severe acute respiratory syndrome coronavirus 2 and influenza virus yielded negative results. Sputum and blood culture results were also negative. The patient did not take medications that can cause drug‐induced pneumonia. He was diagnosed with acute exacerbation of chronic interstitial pneumonia and was, therefore, treated with methylprednisolone (500 mg/day) upon admission (Figure 2). However, on the fourth day of admission, his serum D‐dimer level increased to 62.46 μg/mL, and contrast‐enhanced CT scan showed multiple contrast defects mainly in the peripheral lower lobe branches of the bilateral pulmonary arteries (Figure 1E). He was then diagnosed with pulmonary thromboembolism, and treatment with fondaparinux 7.5 mg/day was initiated. After the initiation of fondaparinux treatment, the patient's serum D‐dimer levels decreased, which led to a gradual reduction in oxygen supplementation. Subsequent laboratory testing revealed positivity for anticardiolipin antibody immunoglobulin M (74.8 U/mL), which indicated that the embolisms might be attributed to APS. On the 7th day of hospitalisation, systemic corticosteroids were switched to prednisolone (PSL) at 60 mg/day and its dose was quickly tapered. On the 11th day of admission, fondaparinux was replaced with oral edoxaban at 60 mg/day. By the 30th day of hospitalisation, the PSL dose was tapered to 10 mg/day, and the patient was discharged. Contrast‐enhanced CT scan performed 1 month after discharge showed improvement in the pulmonary thrombus (Figure 1F). Subsequent laboratory testing conducted after 2 months revealed an elevated serum anticardiolipin antibody IgM level (61.9 U/mL). Contrast‐enhanced CT revealed multiple bilateral pulmonary thrombi, and serum anticardiolipin IgM antibodies were detected on two occasions at least 12 weeks apart, fulfilling the diagnostic criteria for APS; therefore, the patient was diagnosed with APS. Corticosteroids were discontinued 97 days after discharge. By 90 days post‐discharge, the patient did not require oxygen therapy and was managed solely with edoxaban. The patient remains under outpatient care.

**TABLE 1:** Laboratory data upon admission.

Parameters	Values
CRP	7.08 mg/dL
TP	6.4 mg/dL
Alb	2.7 g/dL
AST	23 IU/L
ALT	8 IU/L
LDH	360 IU/L
WBC	7135/μL
RBC	338 × 10^4^/μL
Hb	7.6 g/dL
Plt	23.5 × 10^4^/μL
D‐dimer	38.97 μg/mL
KL‐6	1236 U/mL
SP‐A	90 ng/mL
ANA	< 40 times
MPO‐ANCA	0.2 U/mL

Abbreviations: Alb, albumin; ALT, alanine aminotransferase; ANA, antinuclear antibodies; AST, aspartate aminotransferase; CRP, C‐reactive protein; Hb, haemoglobin; KL‐6, Krebs von den Lungen‐6; LDH, lactate dehydrogenase; MPO‐ANCA, myeloperoxidase‐specific antineutrophil cytoplasmic antibodies; Plt, platelet; RBC, red blood cell; SP‐A, surfactant protein A; TP, total protein; WBC, white blood cell.

## 3 Discussion

This case emphasises two important clinical points: First, pulmonary thromboembolism caused by APS can mimic IP‐AE. Second, early initiation of anticoagulation with systemic corticosteroids is essential in such a case.

The pulmonary manifestations of APS can resemble IP‐AE on imaging, particularly in the form of bilateral ground‐glass opacities. Previous reports have shown cases resembling IP‐AE, as summarised in Table 2 [3, 7, 8, 9]. There have been five reported cases of APS with pulmonary lesions mimicking IP‐AE, including our case. In all cases, the chest CT scan findings revealed bilateral ground‐glass opacities and reticular shadows. All patients were treated with systemic corticosteroids and four of them with anticoagulant drugs. Two patients, including our patient, received anticoagulant therapy immediately after hospitalisation and survived. However, the other three patients, in whom anticoagulant therapy was delayed or not administered, died. Two patients had elevated serum D‐dimer and fibrin degradation product levels. However, the other three had no available data. In addition, pathological lung findings were described in three of the four patients. Multiple thrombi in the smaller veins and fibrotic intimal thickening were identified in one of these three patients. The remaining two patients exhibited findings indicative of interstitial pulmonary fibrosis and diffuse alveolar damage or thickened alveolar septa due to diffuse but variable fibrosis and inflammation, with numerous hemosiderin‐laden macrophages in the alveolar spaces. However, thrombi were not identified. In this case, although pathological findings were not obtained, multiple thrombi were identified on CT scan imaging. Therefore, APS mimicking acute exacerbation of IP is unlikely to represent a single clinicopathologic entity. Accordingly, due to their anti‐inflammatory effects, corticosteroids should be considered along with anticoagulation therapy.

**TABLE 2:** Clinical characteristics of four cases of antiphospholipid antibody syndrome requiring differentiation from interstitial pneumonia.

	Kelion et al. [3]	Savin et al. [7]	Minatani et al. [8]	Kameda et al. [9]	Current case
Age/sex	58/Female	41/Male	53/Male	49/Female	77/Male
Diagnosis	APS and IPF, followed by pulmonary thromboembolism (postmortem)	APS with interstitial pneumonia (postmortem)	SSc‐associated ILD with APS (postmortem)	APS that progressed to CAPS in the context of MCTD overlap	APS, followed by pulmonary thromboembolism and subsequent ARDS
Imaging findings	CXR: bilateral lower lobe reticular shadows	CXR: cardiomegaly, bilateral alveolar infiltrates; CT scan: GGO	CXR/CT scan: bilateral reticular shadows	CT scan: mild GGO and linear shadows in the bilateral lower lobes	CT scan: bilateral ground‐glass opacities
Treatment	Corticosteroids, heparin	Warfarin, corticosteroids	Corticosteroids, aspirin, and heparin	Corticosteroids (discontinuation of anticoagulant therapy)	Corticosteroids, fondaparinux, and edoxaban
Time to anticoagulant initiation	After 6 weeks	On the day of visit	After 2 weeks	Not administered	After 4 days
Outcome	Death during the tapering of corticosteroid dose	Improvement	Death	Death	Improvement
D‐dimer/FDP level (μg/mL)	Data not available	Data not available	Data not available	FDP: 18	D‐dimer: 38.97
Pathological lung finding	None	Pulmonary fibrosis with inflammation	Diffuse alveolar damage (DAD)	Venous thrombi with intimal fibrosis	None

Abbreviations: APS, antiphospholipid syndrome; ARDS, acute respiratory distress syndrome; CAPS, catastrophic antiphospholipid syndrome; CT, computed tomography; CXR, chest radiography; FDP, fibrin degradation products; GGO, ground‐glass opacity; IPF, idiopathic pulmonary fibrosis; MCTD, mixed connective tissue disease; SSc‐ILD, systemic sclerosis‐associated interstitial lung disease.

To establish an APS diagnosis, the presence of antiphospholipid antibodies on two occasions, at least 12 weeks apart, in addition to thrombotic symptoms, should be confirmed. However, in patients who survived, anticoagulant drugs were initiated based on elevated serum D‐dimer levels or a single positive examination finding of antiphospholipid antibodies because APS was suspected. These findings indicate that APS should be included as a differential diagnosis of IP‐AE and that treatment with anticoagulants must be initiated as early as possible. In the current case, the patient exhibited elevated serum D‐dimer levels. Hence, contrast‐enhanced chest CT scan was performed, which revealed pulmonary thrombus in an area consistent with pulmonary shadows, leading to the diagnosis of pulmonary thromboembolism. Therefore, in patients suspected of IP‐AE, the possibility of APS‐related pulmonary lesions should be considered, and appropriate diagnostic testing must be performed.

In the current case, the combination of corticosteroids and anticoagulants in the initial phase, followed by anticoagulants in the maintenance phase, was effective. CAPS, a severe type of APS, causes acute respiratory distress syndrome [10, 11]. Although several mechanisms have been proposed for CAPS‐related acute respiratory distress syndrome, its exact pathophysiology remains unclear [10]. It is believed that systemic intravascular thrombosis, inflammatory responses, increased vascular permeability and complement activation interact in a complex manner, leading to impaired microvascular blood flow and inflammatory lung injury [10, 11]. The treatment strategies for CAPS include anticoagulation therapy, corticosteroids, high‐dose intravenous immunoglobulin and plasma exchange [6, 11, 12]. In the current case, anticoagulation and systemic corticosteroid treatment were effective. However, Systemic corticosteroids may be required at least until a definitive diagnosis is established or a good response to anticoagulant drugs is confirmed, because CAPS with severe lung involvement may mimic IP‐AE. In addition, if the treatment response is inadequate, additional strategies such as intravenous immunoglobulin and plasma exchange should be considered.

Although there are only a few reported cases of APS mimicking IP‐AE, its prevalence could be underestimated. Thus, IP‐AE should be distinguished from APS. Moreover, further case accumulation and a more detailed understanding of the underlying pathology are needed.

## Author Contributions

Saki Ishii: writing – original draft; data curation. Hiroki Wakabayashi: writing, review and editing. Kazutoshi Isobe: project administration; conceptualisation, writing, review and editing. Ryogo Ohashi, Kensuke Namba, Misa Iwayanagi, Hiromasa Sakurai, Daiki Sakai, Kenta Takashima, Yu Murakami, Kaichi Kaneko, Yasuo Matsuzawa: data curation.

## Funding

The authors have nothing to report.

## Consent

The authors declare that written informed consent was obtained from the patients for the publication of this manuscript and accompanying images using the form provided by the Journal.

## Conflicts of Interest

The authors declare no conflicts of interest.

## Data Availability

Data sharing not applicable to this article as no datasets were generated or analysed during the current study.
